# ‌A century-scale dataset of crop yield and agronomic trait evolution under field management in China

**DOI:** 10.1038/s41597-026-07545-0

**Published:** 2026-06-08

**Authors:** Xinyu Ding, Bingfu Yao, Yaping Huang, Xue Liu, Xin Li, Shaomin Huang, Wenju Zhang

**Affiliations:** 1 State Key Laboratory of Efficient Utilization of Arable Land in China, Key Laboratory of Arable Land Quality Monitoring and Evaluation, Ministry of Agriculture and Rural Affairs/Institute of Agricultural Resources and Regional Planning, Chinese Academy of Agricultural Sciences, Beijing, 100081 China; 2 Institute of Plant Nutrition and Environmental Resources Science, Henan Academy of Agricultural Sciences, Zhengzhou, 450002 China

## Abstract

We present a century-scale dataset on crop yield and agronomic trait evolution under field management in China. This resource compiles 22,017 records from 802 sites and 504 studies conducted across China over the past century, with a primary focus on long-term experiments. The dataset covers five major crops: rice, wheat, maize, soybean, and rapeseed. It includes 56 variables, such as crop yields, agronomic traits, field management practices (including fertilizer application, tillage, and crop rotation), and variety characteristics. Maximum potential yield for each variety under ideal conditions is also provided. Each record is georeferenced and time-specific, supporting analyses of yield trends and impacts of management or breeding strategies. This dataset enables detailed investigation of the temporal evolution of soil fertility and yield gaps. It serves as a robust foundation for crop yield modeling, sustainability assessment, and food security research. The data effectively complements existing statistical and remote sensing resources.

## Background and Summary

Cereal and oilseed production underpin national security and public welfare. China is responsible for feeding approximately 17% of the global population while possessing only 7% of the world’s arable land, making the understanding and improvement of crop yields critically important. In 2024, China’s per capita grain availability reached 493 kg, representing a 2.4-fold increase since 1949^1^. Although grain availability and overall food supply have increased substantially, full self-sufficiency remains elusive, and future demand is projected to rise. The total food supply quantity is currently 1085.9 kg capita^−1^ year^−1^ (FAO, 2023)^2^, and Stehl *et al*. (2025) confirm that China remains short of full self-sufficiency^3^. As the Chinese population and living standards increase, grain demand is expected to rise further due to higher consumption of both staples and non-staple foods, including fruits, vegetables, dairy, and meat^1–3^. This trend highlights the urgent need for comprehensive, integrated datasets that link crop yields with detailed field management and varietal information. These datasets are essential for assessing yield potential, identifying yield gaps, and informing sustainable intensification strategies.

Changes in China’s crop production will have significant implications for global food supply chains^3–5^. Enhancing field management and optimizing crop varieties are fundamental strategies for achieving sustainable yields growth and food security^1,5^. Integrated datasets that combine crop yield, field management, and varietal information are essential for quantifying production potential and yield gaps^2,6^. Such datasets form a robust foundation for model development, validation^7^_,_ and the formulation of targeted agricultural policies^8,9^. In China, reconstructing historical yield and management practices is challenging due to fragmented and inaccessible data sources. Prior to 1980, records were limited and often employed inconsistent measurement units, reducing their usefulness. Although data availability increased after 1980, high-quality observations from long-term field experiments remain lacking. Consequently, the long-term impacts of field management and varietal changes on crop yields are insufficiently quantified due to the absence of harmonized, long-duration datasets. Addressing these deficiencies is critical for advancing modeling efforts, policy development, and scientific understanding of yield trends and sustainable food production in the context of evolving climatic and socio-economic conditions. The primary objective of this dataset is to establish a unified, accessible, and comprehensive resource that integrates yield and agronomic data from over a century of field experiments, thereby supporting rigorous scientific analysis and evidence-based decision-making.

Advancements in field management techniques, including fertilizer application, agricultural mechanization, and engineering measures, together with the development of improved crop varieties, have increased cereal and oilseed yields in China by 6.2-fold between 1949 and 2024^1^. Numerous datasets on spatial yield generation have been compiled from provincial and municipal statistics over the past 40 years by sources such as the National Bureau of Statistics of China^10,11^ and agricultural yearbooks^6^. These datasets offer long-term perspectives on the spatial distribution and productivity of these crops, reflecting the combined effects of economic, policy, and environmental factors. Nevertheless, detailed yield-related information, particularly field management practices and variety changes, remains limited and requires systematic data derived from long-term field experiments. Experiments that control variables such as fertilizer, pesticide, and herbicide application, tillage, crop rotation, and irrigation are less affected by economic and policy fluctuations. As a result, century-long datasets of yields and agronomic traits from field experiments on cereal and oilseed crops provide essential supplements to statistical and remote sensing datasets, facilitating comprehensive analysis of yield trends and agronomic impacts.

Scientific investigations into nutrient input and variety improvement in China have been conducted for more than a century^12–14^. Early fertilizer research began at the Gongzhuling Agricultural Experiment Station in 1913^13,14^. Subsequently, China established a nationwide collaborative experiment network, which expanded significantly during the 1930s, 1950s, and 1970s^12,15^. This dataset collects all yield-related data from these historical experiments conducted before 1980. However, the relationship between environmental factors and crop yields is complex and evolves slowly. To reliably assess the effects of field management practices and crop variety improvements on yield, it is essential to use representative data that are long-term and consistently accumulated, ensuring robustness and accuracy. After 1980, many national experiments in collaboration shifted to long-term *in situ* field studies, while local universities and research institutes initiated new yield-related experiments. During the same period, China reformed its variety approval system in the 1980s^16^, making data from both regional testing and production trials publicly available. Consequently, this dataset includes yield-related data from long-term field experiments and variety approvals conducted after 1980.

Field trials remain fundamental for evaluating the relative contributions of management practices and crop variety improvements to yield growth^17–19^. The dataset aims to compile over a century of crop yield data into a unified resource to support global research on crop productivity in China. It contains more than 20,000 yield-related records extracted from 504 peer-reviewed studies conducted between 1913 to 2025. The dataset includes field management factors (fertilizer application rate, tillage methods, crop type, and rotation), crop varieties (name and yield of approved varieties), yields (grain and straw), and agronomic traits (crop height, thousand kernel weight, phenological stage, harvest index). This comprehensive dataset provides a valuable foundation for modeling studies focused on yield prediction, identifying yield gaps, and validating model outputs and empirical findings.

## Methods

### Data collection

**Fig. 1:**
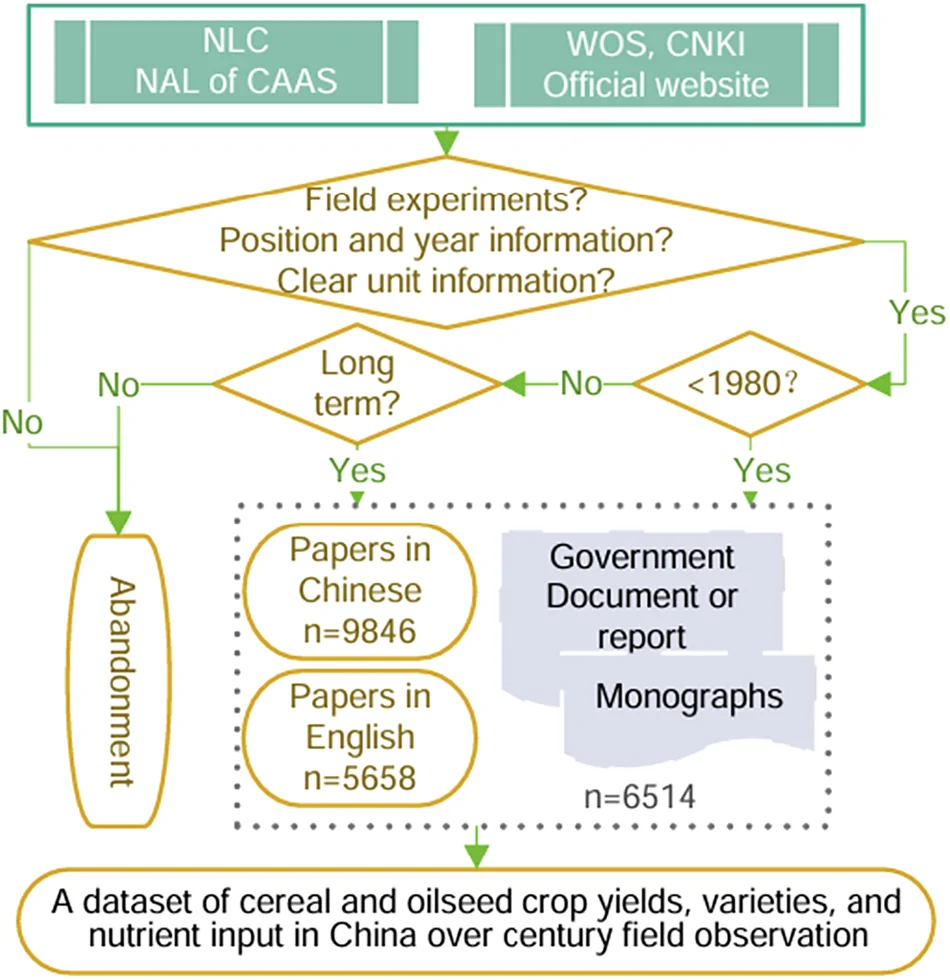
Data processing approach for data collection, screening, and integration of the dataset.

Strict criteria were applied to collect data from both libraries and websites (Fig. 1), covering the period from 1913 to May 2025. Observations (n = 974) on crop yields and fertilizer rates published before 1949 were accessed at the National Library of China^12^. Data from monographs (n = 4,008) and government documents (n = 1,956) were obtained through the National Agricultural Library of the Chinese Academy of Agricultural Sciences. Permanent links for book searches are available in the “Reference” sheet and may be used to borrow books from the closed-stack collection if necessary.

For literature published after 1949, Chinese papers were obtained from China National Knowledge Infrastructure (http://www.cnki.net), while English papers were collected from Web of Science (https://www.webofscience.com/wos/alldb/basic-search). We use the search keywords “Yield” Or “Production” AND “Maize” Or “Wheat” Or “Rice” Or “Soybean” Or “Rapeseed” to conduct our research. Since 1980, research on crop yields has increased rapidly and the data volume became too large to collect comprehensively. During this period, many long-term field experiments were established in China. To ensure data quality, we incorporated the keyword “Long term” to select high-quality yield data from field studies lasting more than three years. DOIs for relevant papers are provided in the “Reference” sheet.

Data from government reports (n = 128) were retrieved from the China Seed Association’s website (http://seedchina.com.cn/). URLs of “High-yielding Variety Yield Test Results” from 2018 to 2024 are included in the “Reference” sheet. Additionally, yield data for variety approval (n = 3,145) were obtained from the Big Data Platform of China Seed Industry (http://202.127.42.145/bigdataNew/). To ensure link accessibility, we have included PDF files (titled “Data_from_government_report.pdf”) containing the downloaded yield data, along with a step-by-step guide for searching crop yield information on the platform^20^.

All included studies and experimental data met the following criteria: (a) field experiments; (b) information on location and year; (c) clear units; and (d) yield and agronomic trait data that could be directly extracted from figures (using GetData Graph Digitizer 2.24), tables, or text.

**Fig. 2:**
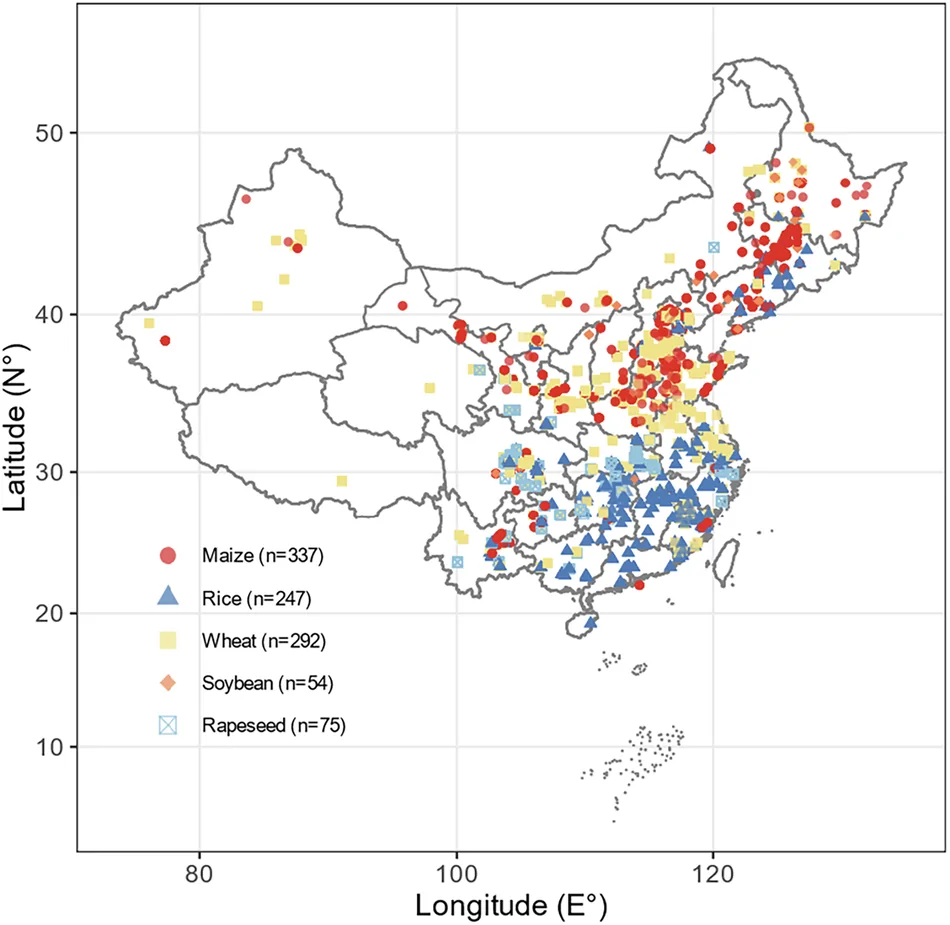
The geographic distribution of sites within the dataset.

**Fig. 3:**
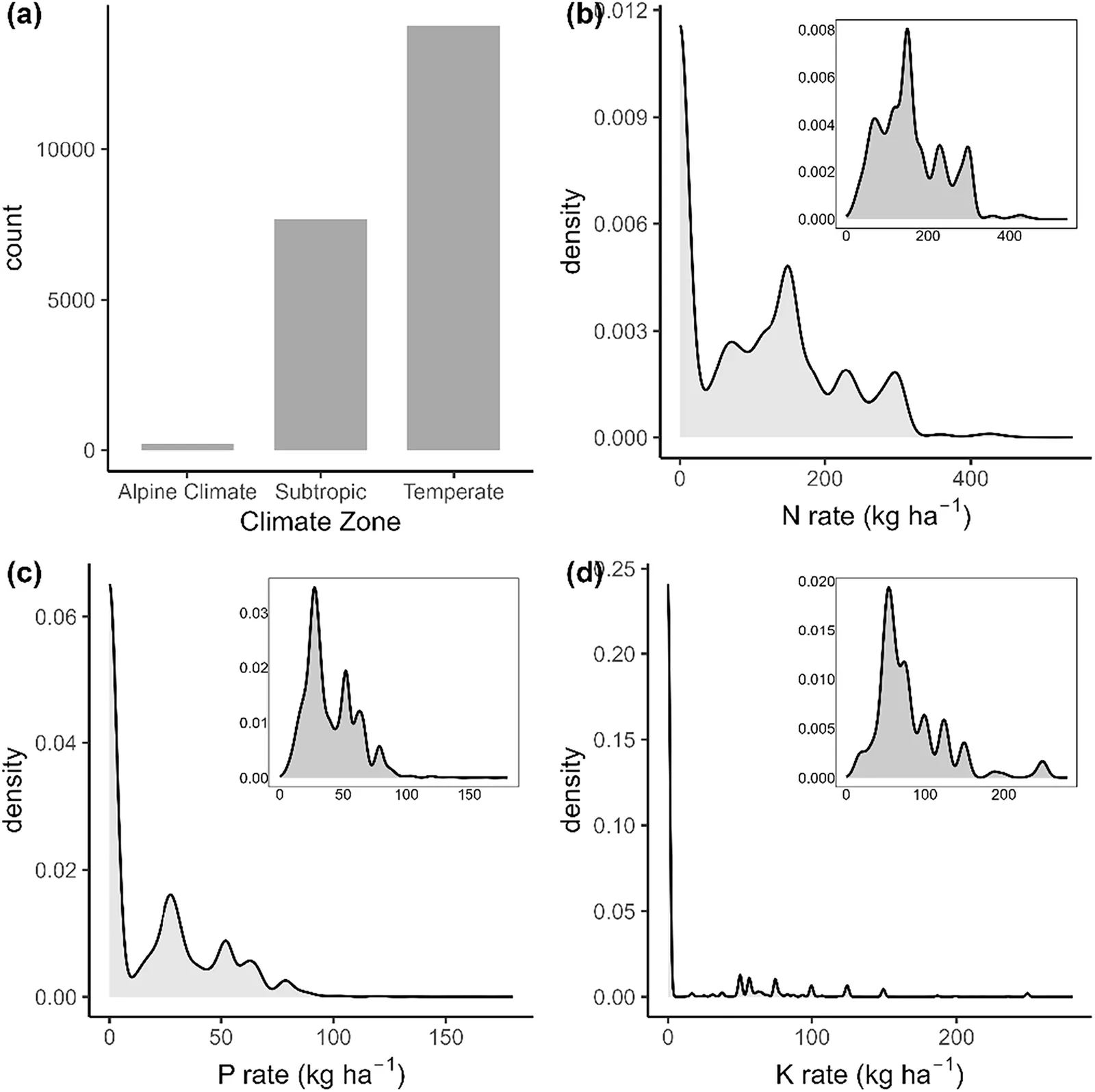
Distribution of samples in the dataset across various climate zones (**a**) and at different application rates of nitrogen (**b**), phosphorus (**c**), and potassium (**d**).

A total of 3,241 field experiments from 504 published studies conducted across 802 sites were compiled. The Site_ID, Study_ID, and Experiment_ID are provided in the “Data” sheet. The spatial distribution of these experimental sites is depicted in Fig. 2. Among the entries, 21,061 included data on types and application rates of chemical fertilizers (nitrogen, phosphorus, and potassium), while 19,721 provided crop yield data (grain and straw) across initial, sampling, and final years. The number of entries containing crop variety information, including crop varieties and yield results from variety approval processes, such as approval year, regional test yields, and production trial yields, is presented in Table 1. For agronomic traits, thousand kernel weights and phenophase information, including sowing (or transplanting for rice) and maturity (harvest) times were collected when reported in the study. In addition to yield and agronomic traits, harvest index and partial factor productivity from fertilizers (nitrogen, phosphorus, and potassium) were extracted or calculated from the selected studies. The dataset also includes information on the first author, publication year, site location (latitude and longitude, Fig. 2), and climate zone (Fig. 3).

**Table 1:** Description and number of the dataset.

Information type	Related index	Maize	Wheat	Rice	Soybean	Rapeseed
Nutrient input	Chemical fertilizer types, application rates, and organic nutrient inputs	6570	7603	4724	1408	756
Crop varieties	Variety of cereal and oilseed crops	1915	1879	1411	949	262
Grain yield	Mean, n, and SD	6380	6817	4451	1380	693
Straw yield	Mean, n, and SD	355	368	487	588	3
Approval yield	Grain yield from variety approval (both regional tests and production trials)	1219	1177	514	164	71
Height	Mean, n, and SD	170	135	103	21	42
Kernel Weight	Mean, n, and SD of thousand kernel weight	355	569	238	32	42
Phenophase information	Sowing and maturity time of crops	1197	2735	1533	72	9

### Data processing

For locations without latitude and longitude data, the geographical coordinates of the corresponding village or county were used as supplementary information. The climate zone of each study site was determined and supplemented using its latitude and longitude coordinates. All yield units in this dataset were standardized to kg ha^−1^. The mean, number of replications (*n*), and standard deviations (SD) were gathered from the publications when possible. If only standard error (SE) or coefficient of variation (CV) were reported, the SD was calculated as follows:

1 $$SD=SE\times \sqrt{n}$$

2 SD=CV×Mean

When publications reported only the mean without SD, SE, or CV, SD was estimated by multiplying the mean by the average coefficient of variation of the entire dataset^21^. Sensitivity analysis indicated that CV values for maize and wheat were significantly affected by stratification based on climate, nutrient management, and tillage measures (*p* < 0.05). Rice was significantly influenced by climate and nutrient management, while soybean and rapeseed were affected only by nutrient management. For cases with missing SD values, CV calculations and SD imputations were conducted by stratifying data according to crop type, climate, nutrient management, and tillage measures.

*n* were missing for 50.4% of the observations (11,096 out of 22,017). The reported *n* values ranged from 3 to 270, with a median of 3 (IQR: 3–4). To address missing *n* values, we evaluated several imputation strategies (Median, Q1, and Q3). As shown in Table 2, the estimated means for rapeseed and rice were consistent across these methods, while Q3 imputation yielded substantially higher means for maize and soybean. To avoid potential overestimation bias, we selected Median imputation as the primary approach. Specifically, when *n* was not reported in the original publication, the median n of the dataset was used for imputation^21^.

**Table 2:** Comparison of estimated means under different missing data imputation strategies across crop types.

Crop type	Median	Q1	Q3	Mean
Maize	3.28	3.28	3.85	3.67
Rapeseed	3.16	3.16	3.16	3.42
Rice	3.85	3.85	3.85	4.58
Soybean	3.04	3.04	3.89	3.33
Wheat	3.66	3.66	4.1	4.19

For studies that did not directly report yield, we estimate yield values using the change in yield rate and rate per unit area for different fertilizers treatments^12,15^, as outlined in the “Calculation” sheet. The Harvest Index (HI) and Partial Factor Productivity of chemical fertilizers (PFPF) were extracted from the publications whenever available. If not reported, they were calculated as follows:

3 $$HI=\frac{{Y}_{G{\rm{rain}}}}{{Y}_{Straw}+{Y}_{Grain}}$$

4 $$PFPF=\frac{{Y}_{Grain}}{{F}_{Application}}$$

Where *Y* is grain yield and *F* represents the chemical fertilizer application rate. The HI was calculated only when both grain and straw yields were available. In certain experiments, residual stubble remaining on the soil surface post-harvest may lead to overestimated HI values. Similarly, PFPF was computed exclusively for treatments with recorded grain yield and chemical fertilizer application. To ensure accurate PFPF assessment, treatments involving organic amendments were omitted from the analysis.

## Data Records

**Fig. 4:**
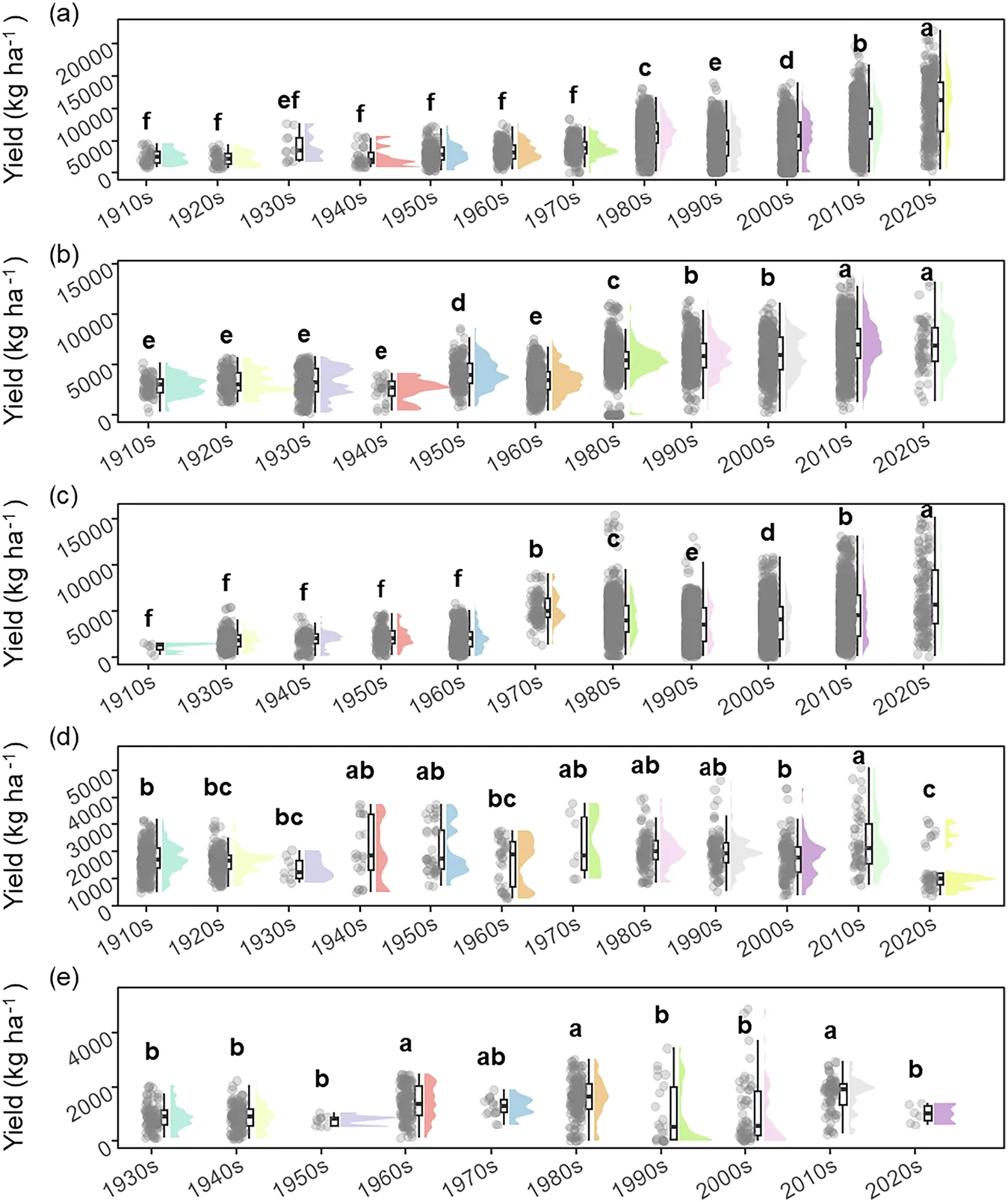
Grain yield dynamics of five major crops from 1910 to 2020 ((**a–e**) represent maize, rice, wheat, soybean, and rapeseed, respectively. Different lowercase letters indicate significant differences among decades according to Tukey’s HSD test (p < 0.05).

**Fig. 5:**
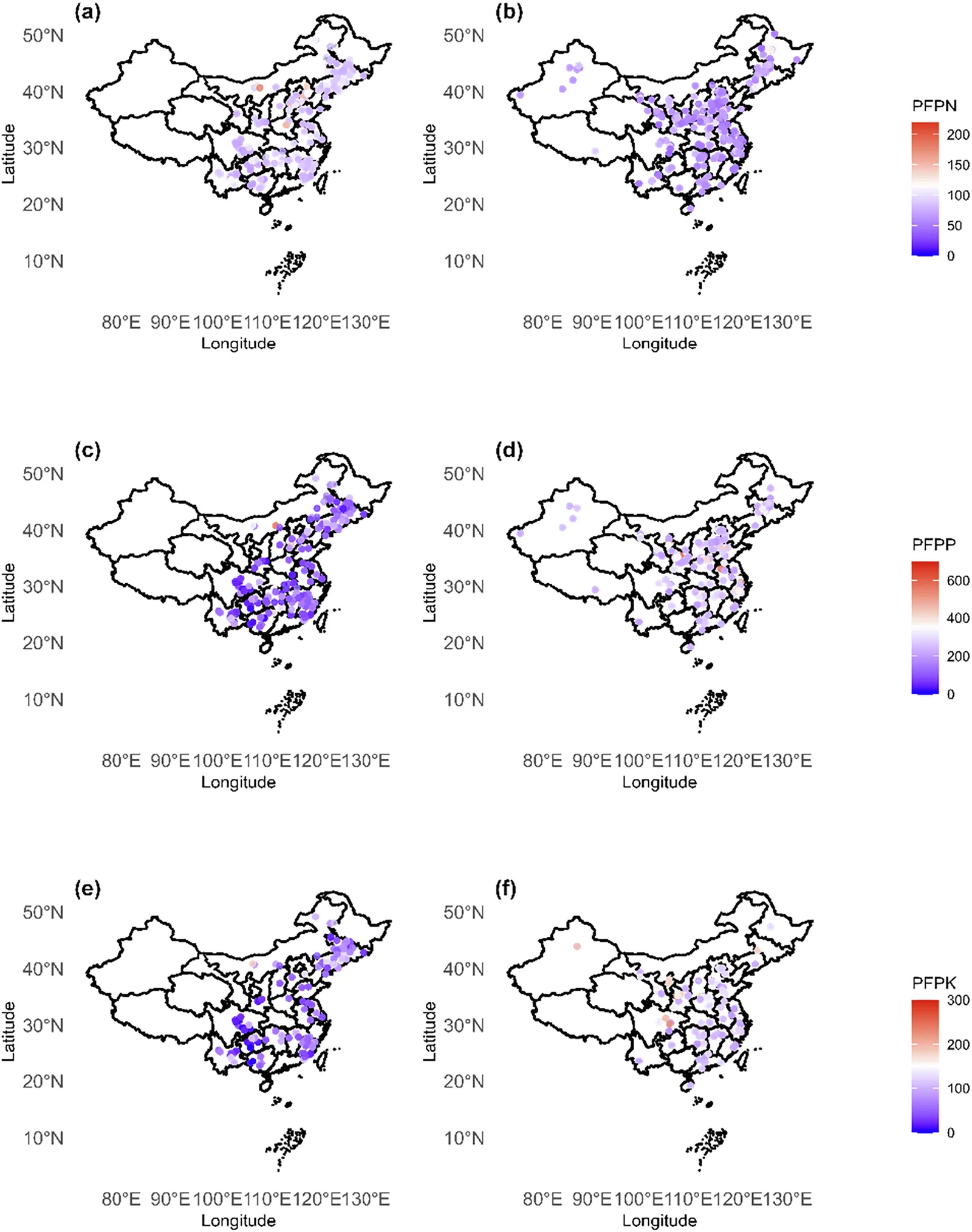
Temporal and spatial modifications in partial factor productivity of nitrogen (**a** and **b**), phosphorus (**c** and **d**), and potassium (**e** and **f**) during 1910s–1970s (**a,** **c**, and **e**) and 1980s-2020s (**b,** **d**, and **f**).

All data and R code are available^20^. The dataset, titled “China_yield_centurial_v1.xlsx”, includes 22,018 rows and 56 columns, derived from 504 studies (listed in the “Reference” sheet). Each row contains study details such as site location, climate zone, crop type, crop varieties, fertilizer type and rate, tillage method, yield, thousand kernel weight, plant height, and other agronomic traits. The “Nutrient” row details organic and inorganic nutrient inputs applied in the field experiments. The number of climate zones and the density of inorganic fertilizer application rates are shown in Fig. 3. Rice, wheat, and maize yields have increased over time (Fig. 4). The PFPN declined from the 1980s–2020s compared to the 1910s–1970s; however, the PFPP and PFPK showed opposite trends (Fig. 5). When searchable via the China Seed Association website, the dataset also includes regional test and production trial grain yields, along with the variety approval year.

The sheet titled “Explanation and Unit” details the data and specifies the units for each standardized indicator. The “Reference” sheet lists the titles of publications and papers in both Chinese and English; many studies published before 1980 were only available in Chinese, so Chinese titles have been retained to ensure dataset traceability. The “Calculation” sheet provides the units commonly used across different study periods and explains the calculation process for yield, including changes in yield amounts and rates per unit area for various fertilizers.

## Technical Validation

The authenticity of the research and original data was confirmed prior to data extraction. Each study was reviewed at least twice, and duplicate studies were subsequently removed. After extraction, all variable distributions were examined, and outliers exceeding biologically plausible limits for each crop type were removed to maintain data quality. Outliers representing unfeasible yields were excluded, defined as data points surpassing the biological maximum for each crop—specifically, values higher than the highest yields documented in official variety approval trials or specialized high-yield experiments (listed in the “Data” sheet). Manual inspection of the data and units was performed to identify potential errors associated with outliers and extreme points. Field experiments showed higher yields compared to statistical and remote sensing datasets, attributable to optimized field management practices. During the 2020 s, soybean yields exhibited significant variability, primarily influenced by data from high-yield field trials and sloping farmland experiments (Fig. 5). Nonetheless, the patterns of temporal and spatial yield variation remained consistent across these methods^22–24^. Meanwhile, the mean partial factor productivity of nitrogen (PFPN) value (40 kg kg^−1^) from the 1980s–2020s is consistent with the previous meta-analysis study^25^.

## Usage Notes

This dataset is intended for researchers examining long-term trends in Chinese agriculture, food security modeling, and the interactions between agricultural practices and environmental changes. While supported by long-term field experiments that underpin its scientific validity and reliability, it should be noted that these experiments in China commenced later than in other countries. Users should be aware of the methodological shift that began in the 1980s, when long-term field experiments were initiated. The initial and end year columns indicate the duration of each experiment; the same location with identical years suggests the same experiment, while changes in crop type during the experiment reflect rotation sequences. Additionally, grain yields from regional testing and production trials used for variety approval since 1980 provide valuable data to assess yield gaps and understand how improvements in field management and crop varieties contribute to yield increases.

Field experiments on crop yields involve controlled practices such as fertilizer application, tillage, and crop rotation, conducted without direct influence from economic or policy factors. The dataset, which encompasses a century of yield records and agronomic traits derived from these experiments, serves as a valuable complement to statistical and remote sensing datasets. It can be used to validate existing yield models and extend their temporal applicability. Utilizing this dataset allows for the assessment of land productivity modifications over the past century. When combined with soil property and climate data, it facilitates the analysis of soil health evolution and enhances the accuracy of evaluating self-sufficiency and agricultural sustainability amid climate change.

## Data Availability

The dataset generated and analysed in this study has been deposited in figshare (https://doi.org/10.6084/m9.figshare.30819320).

## Code availability

Data visualization was performed using R (version 4.2). The script “China_yield_centurial_v1.R” includes code for plotting site locations (Fig. 2), analyzing variable distributions (Fig. 3), yields (Fig. 4), and the partial factor productivity of chemical fertilizers (Fig. 5). All code and data are available for access^20^.
